# Classification of rice leaf blast severity using hyperspectral imaging

**DOI:** 10.1038/s41598-022-22074-7

**Published:** 2022-11-17

**Authors:** Guosheng Zhang, Tongyu Xu, Youwen Tian, Shuai Feng, Dongxue Zhao, Zhonghui Guo

**Affiliations:** Shenyang Agricultural University, Shenyang, China

**Keywords:** Computational biology and bioinformatics, Plant sciences

## Abstract

Rice leaf blast is prevalent worldwide and a serious threat to rice yield and quality. Hyperspectral imaging is an emerging technology used in plant disease research. In this study, we calculated the standard deviation (STD) of the spectral reflectance of whole rice leaves and constructed support vector machine (SVM) and probabilistic neural network (PNN) models to classify the degree of rice leaf blast at different growth stages. Average accuracies at jointing, booting and heading stages under the full-spectrum-based SVM model were 88.89%, 85.26%, and 87.32%, respectively, versus 80%, 83.16%, and 83.41% under the PNN model. Average accuracies at jointing, booting and heading stages under the STD-based SVM model were 97.78%, 92.63%, and 92.20%, respectively, versus 88.89%, 91.58%, and 92.20% under the PNN model. The STD of the spectral reflectance of the whole leaf differed not only within samples with different disease grades, but also among those at the same disease level. Compared with raw spectral reflectance data, STDs performed better in assessing rice leaf blast severity.

## Introduction

Rice, a major grain crop worldwide, accounts for approximately one-quarter of the total crop planting area in China and one-third of the grain yield^1^. Rice blast caused by *Magnaporthe grisea* is one of the most severe rice diseases in China^2^ and is responsible for serious economic losses. Beginning in the 1990s, the average annual occurrence area of rice blast in China has been at least 3.8 million hm^2^, with annual losses of up to several hundred million kilograms^3^. To improve rice productivity and facilitate precision agriculture, accurate evaluation of rice blast severity is therefore of great importance.

Current disease scouting and phenotyping techniques mostly depend on human visual ratings^4–6^. In these approaches, the eye functions as a remote sensing device and acts in combination with relevant parts of the brain as an image analysis system^7^ able to rapidly capture, analyse and comprehend images^8^. In this way, individuals can determine the type and quantity of diseased tissue on a particular leaf or plant^9^. Human visual ratings rely on rater capacity and credibility and can be prone to human inaccuracy, subjectivity, and inter/intra-rater variability^10–12^. A novel, more stable method is thus needed.

Hyperspectral imaging is an emerging means of assessing plant vitality, stress parameters, nutrition status, and diseases^13^. Hyperspectral imaging can overcome the aforementioned shortcomings of visual approaches and produce digital measurements that can be easily shared and quickly analysed with semi-automated procedures in a repeatable, objective manner^14^. Compared with multispectral data, hyperspectral data include hundreds of narrow wavebands containing more information^15^ on spatial and spectral^16^ features of rice leaves. Hyperspectral imaging has been used to assess plant disease severity in crops such as wine grapes, barley, and sugar beets^13,16,17^. Data processing methods applied in previous studies, such as analysis of raw spectra^18,19^, difference spectra^20,21^, ratio spectra^22^, derivative spectra^23,24^, and vegetation indices^25–27^, have achieved good results. To our knowledge, however, the standard deviation (STD) of spectral reflectance has not been used for grading rice leaf blast severity.

In this study, hyperspectral images of rice leaves were obtained with a ground-based hyperspectral imaging system, and the average spectral reflectance of whole leaves and different leaf regions were extracted using ENVI 5.6. The STD of spectral reflectance of whole leaves was also calculated automatically using ENVI 5.6. To avoid the possibility of occasionality, full-spectrum-based support vector machine (SVM) and probabilistic neural network (PNN) models were constructed using the STD dataset at three different growth stages. The classification ability of each model was evaluated on the basis of overall classification accuracy and micro and macro F1 values. All values were compared with those of the raw spectra, which were treated as a reference.

## Results

### Spectral characterisation of leaves with different types of lesions

**Figure 1:**
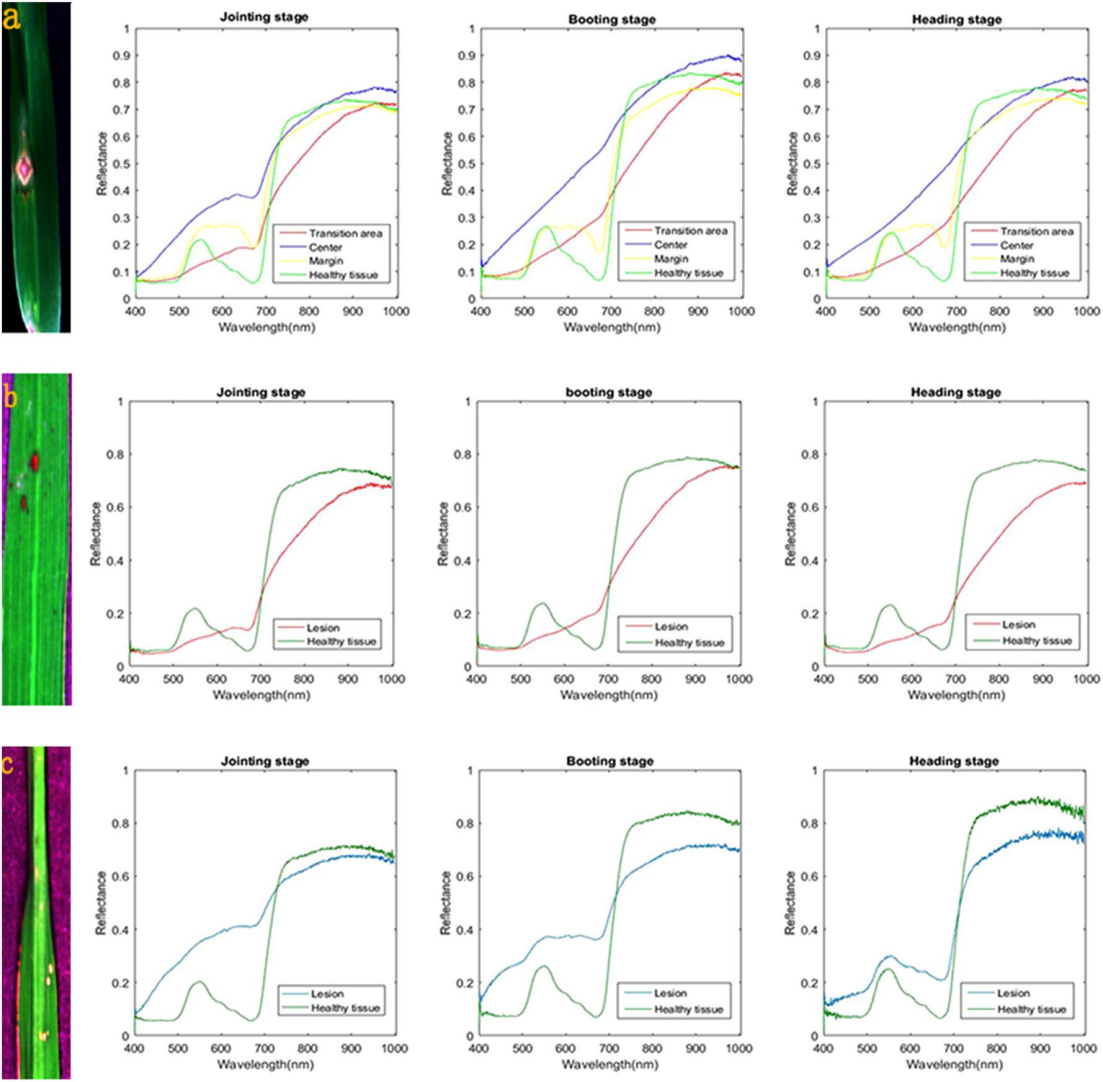
Spectral reflectance of leaves with different forms of lesions at different growth stages. (**a**) The chronic form; (**b**) the brown spot form; (**c**) the white spot form.

Leaves with different types of lesions had hyperspectral images that varied in colour and structure, and their spectral reflectances were also different (Fig. 1). Chronic lesions consisted of three parts: a yellow margin, a red transition area, and a white centre. The spectral reflectance at the centre of these lesions was higher than that of the transition area and the margin across all spectral regions as well as in healthy tissues in most regions. In the visible region, the spectral reflectance of margins was higher than that of healthy tissues, whereas the opposite was true in the near-infrared (NIR) region (Fig. 1a). Brown-spot lesions only comprised a red centre. The spectral reflectance of healthy tissues was higher than that of these diseased areas throughout the whole spectral region, except for 600–700 nm (Fig. 1b). The white-spot form also consisted solely of a centre, but its colour was white. The spectral reflectance in the visible region was lower in healthy tissues than that of white spots, whereas the opposite was true in the NIR region (Fig. 1c).

### Differences in reflectance between diseased and healthy leaves

**Figure 2:**
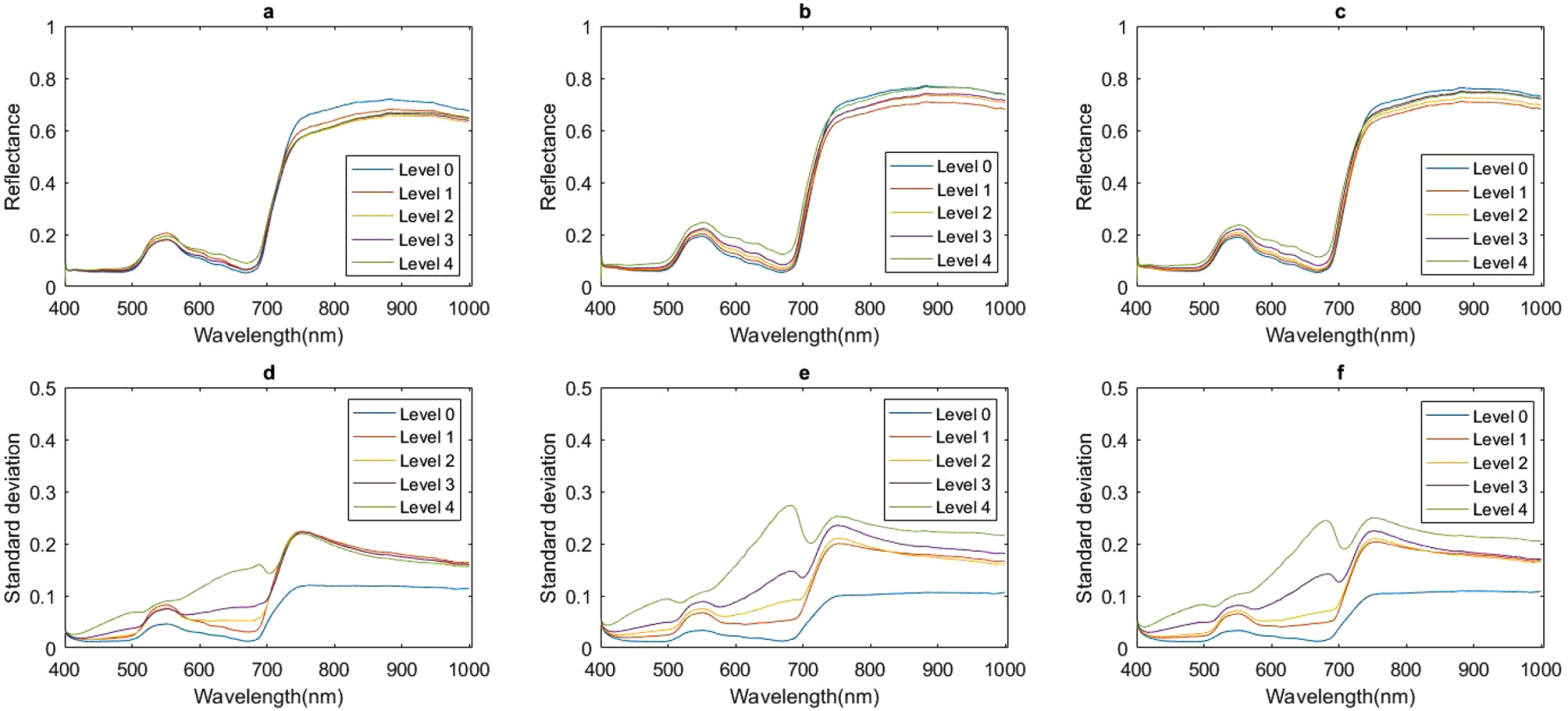
Average spectral reflectance and STD of leaves with different disease degree. (**a**) Average spectral reflectance at jointing stage; (**b**) average spectral reflectance at booting stage; (**c**) average spectral reflectance at heading stage; (**d**) average STD at jointing stage; (**e**) average STD at booting stage; (**f**) average STD at heading stage.

**Figure 3:**
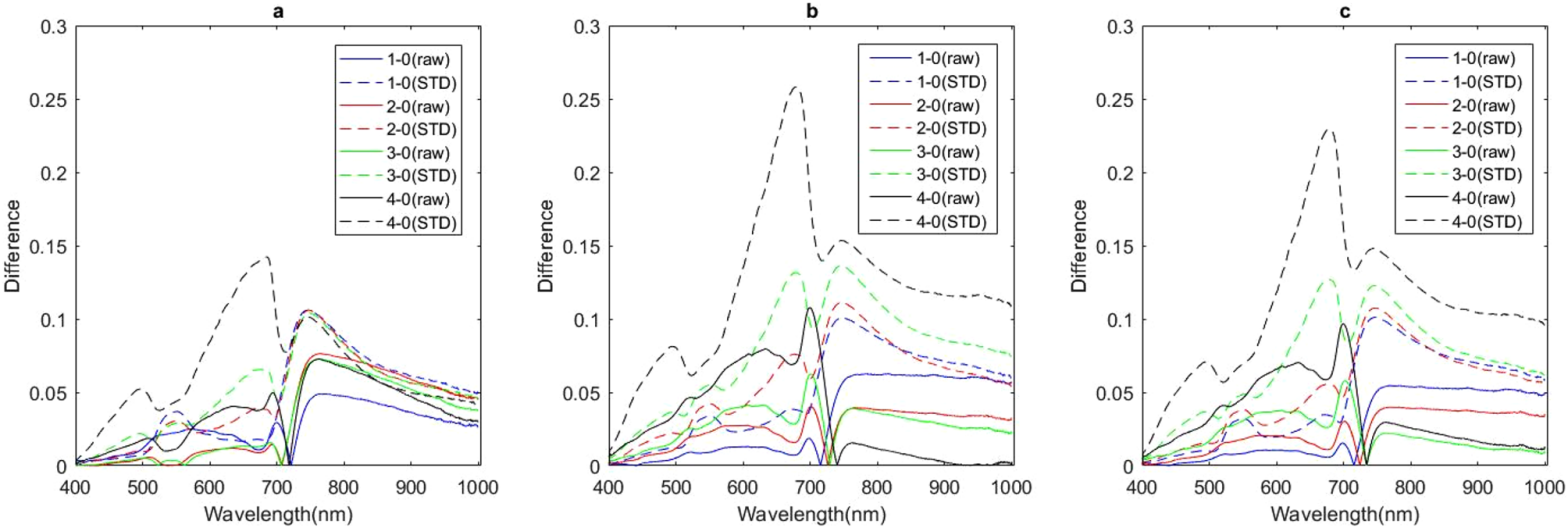
Difference spectra of diseased and healthy leaves at different stages. (**a**) Jointing stage; (**b**) booting stage; (**c**) heading stage.

Some differences in raw spectral reflectance were observed among leaves at five disease levels at three growth stages, but the differences were not obvious. Differences in STD were more evident (Fig. 2). In the visible region, differences in raw spectral reflectance between diseased leaves and healthy ones were positive, and these differences increased with increasing disease severity. In the NIR region, in contrast, differences in raw spectral reflectance between diseased and healthy leaves were negative, and the absolute values of these differences increased with increasing disease severity. The intersection of the difference spectra and the y-axis gradually shifted to longer wavelengths with increasing disease severity. Differences in STD between diseased and healthy leaves were positive throughout the entire spectral region and increased with increasing disease severity (Fig. 3).

### Assessment of rice leaf blast severity using a SVM

To assess the performance of the SVM model, all samples were divided into a training set and a testing set in a ratio of 7:3. This classification model performed well in assessing rice leaf blast severity (Table 1). All three classification measures based on the STD dataset were higher than those derived from raw spectral reflectance data at all three growth stages. Generally, misclassification occurred between adjacent disease degrees (Table 2). Samples were prone to being misclassified as samples of level 1 and level 3.

**Table 1:** Measures for SVM models at different stages.

Growth stage	Average accuracy		Micao F1 value		Macro F1 value	
	Raw (%)	STD (%)	Raw	STD	Raw	STD
Jointing stage	88.89	97.78	0.7222	0.9444	0.7647	0.9497
Booting stage	85.26	92.63	0.6316	0.8158	0.5583	0.7153
Heading stage	87.32	92.20	0.6829	0.8048	0.5974	0.8006

**Table 2:** Performances of the SVM models at different growth stages.

Growth stage	Sample	Raw spectral reflectance						Standard deviation					
		Level 0	Level 1	Level 2	Level 3	Level 4	**True**	Level 0	Level 1	Level 2	Level 3	Level 4	**True**
Jointing stage	Level 0	3	0	0	0	0	3	3	0	0	0	0	3
	Level 1	1	5	0	0	0	6	0	6	0	0	0	6
	Level 2	0	1	2	2	0	5	0	0	5	0	0	5
	Level 3	0	0	1	2	0	3	0	0	1	2	0	3
	Level 4	0	0	0	0	1	1	0	0	0	0	1	1
	**Prediction**	4	6	3	4	1	18	3	6	6	2	1	18
Booting stage	Level 0	2	4	0	0	0	6	6	0	0	0	0	6
	Level 1	0	13	0	1	0	14	0	14	0	0	0	14
	Level 2	0	3	0	1	0	4	0	4	0	0	0	4
	Level 3	0	3	0	5	1	9	0	2	0	7	0	9
	Level 4	0	0	0	1	4	5	0	0	0	1	4	5
	**Prediction**	2	23	0	8	5	38	6	20	0	8	4	38
Heading stage	Level 0	2	2	0	0	0	4	4	0	0	0	0	4
	Level 1	2	12	0	0	0	14	1	12	1	0	0	14
	Level 2	0	6	0	0	0	6	0	2	3	1	0	6
	Level 3	0	2	0	7	1	10	0	1	1	7	1	10
	Level 4	0	0	0	0	7	7	0	0	0	0	7	7
	**Prediction**	4	22	0	7	8	41	5	15	5	8	8	41

### Assessment of rice leaf blast severity using a PNN

For this analysis, all samples were first divided into a training set and a testing set in a ratio of 7:3. The PNN classification model performed well in assessing rice leaf blast severity, although slightly worse than the SVM model (Tables 1 and 3). As in the SVM model, all three STD-based measures were higher than those obtained from raw spectral reflectance at each of the three growth stages. Misclassification mostly occurred between adjacent disease degrees (Table 4). Samples were prone to being misclassified as samples of level 1 at all three growth stages and as level 0 at the heading stage.

**Table 3:** Measures for PNN models at different stages.

Growth stage	Average accuracy		Micao F1 value		Macro F1 value	
	Raw (%)	STD (%)	Raw	STD	Raw	STD
Jointing stage	80	88.89	0.5	0.7222	0.5927	0.7964
Booting stage	83.16	91.58	0.5789	0.7895	0.4828	0.8245
Heading stage	83.41	92.20	0.5854	0.8048	0.5852	0.7625

**Table 4:** Performances of the PNN models at different growth stages.

Growth stage	Sample	Raw spectral reflectance						Standard deviation					
		Level 0	Level 1	Level 2	Level 3	Level 4	**True**	Level 0	Level 1	Level 2	Level 3	Level 4	**True**
Jointing stage	Level 0	1	2	0	0	0	3	3	0	0	0	0	3
	Level 1	1	4	0	1	0	6	0	6	0	0	0	6
	Level 2	0	2	1	2	0	5	0	3	2	0	0	5
	Level 3	0	0	1	2	0	3	0	1	1	1	0	3
	Level 4	0	0	0	0	1	1	0	0	0	0	1	1
	**Prediction**	2	10	2	3	1	18	3	10	3	1	1	18
Booting stage	Level 0	0	6	0	0	0	6	6	0	0	0	0	6
	Level 1	0	13	0	1	0	14	0	14	0	0	0	14
	Level 2	0	3	1	0	0	4	0	2	1	1	0	4
	Level 3	0	2	2	5	0	9	0	0	3	6	0	9
	Level 4	0	0	0	2	3	5	0	0	0	2	3	5
	**Prediction**	0	24	3	8	3	38	6	16	4	9	3	38
Heading stage	Level 0	3	1	0	0	0	4	4	0	0	0	0	4
	Level 1	2	9	0	3	0	14	1	13	0	0	0	14
	Level 2	0	3	2	1	0	6	4	1	1	0	0	6
	Level 3	0	1	2	5	2	10	0	0	1	9	0	10
	Level 4	0	0	0	2	5	7	0	0	0	1	6	7
	**Prediction**	5	14	4	11	7	41	9	14	2	10	6	41

## Discussion

In this study, the STD of whole leaf reflectance was calculated and used for grading rice leaf blast severity at three growth stages. According to our results, performances of the models applied to the STD dataset were significantly better than those obtained using raw spectral reflectance, and rice leaf blast severity could be classified using the STD of spectral reflectance of whole leaves. The proposed data processing method was derived from physiological phenomena visible to the unaided eye, thus making our approach more intuitive and convincing.

In theory, four types of lesions occur under field conditions, namely, acute, chronic, white-spot, and brown-spot forms^28^. Only the latter three forms were observed in this study, however, as acute lesions can be transformed into chronic ones under suitable conditions^28^. Different forms of lesions have diverse effects on leaves. For example, chronic lesions produce conidia, but the other types do not^28^. Non-spore-producing lesions have no effect on future potential disease cycles, thus complicating prediction of the subsequent pathological process. As a result, assessment of rice leaf blast prior to the present study has focussed on a single disease cycle rather than the continual process.

Our spectral characterisation of rice leaf blast revealed that reflectance varied not only within a single lesion, but also among different types of lesions. Reflectance was extremely high in the centre of chronic lesions, a result possibly due to the degradation of photosynthetic pigments within this area^29^. Steinkamp et al.^29^ identified a boundary zone separating diseased from healthy tissue and divided this area into inner and outer regions. Leucker et al.^30^ mapped the two regions to transition areas and margins. Although conducted to investigate sugar beet Cercospora leaf spot (CLS), both of these studies provide a reasonable explanation for the phenomenon observed in the present work. Oerke et al.^21^ also characterised the spectral signatures of healthy tissue and CLS lesions of five sugar beet genotypes. In that investigation, differences in the CLS resistance of diverse genotypes were responsible for the differing spectral signatures of lesions, whereas spectral differences in our study were due to different forms of lesions under the same level of resistance. Despite the similar spectral reflectance profiles uncovered in the two studies, the pathogenic mechanisms of the two diseases (leaf blast and CLS) may be drastically different.

In the STD-based difference spectra, we observed a transition in peak locations from mild (levels 1 and 2) to moderate (level 3) to severe (level 4) disease. In particular, spectra of mildly diseased samples had three peaks located at 551, 675, and 747 nm. The moderately diseased samples exhibited four peaks: one at 495 nm, and the other three the same as in mild disease. The peak pattern of severely diseased samples was identical to that of moderately diseased samples except that the peak at 551 nm was absent. Interestingly, the peaks displayed by both mildly and severely diseased samples were also found in the spectra of moderately diseased samples. We have no explanation for this phenomenon. In contrast to the STD-based difference spectra, the spectra based on raw spectral reflectance only exhibited a single peak, at 700 nm, at all disease levels and growth stages.

The PNN classifier, a type of artificial neural network, simulates the learning mode of the human brain to optimise the clustering centre; however, this adjustment is still affected by the data dimension. When the distribution of data in the dimensional space does not have obvious rules, the selection of clustering centres will be negatively affected. The pure SVM classifier combines spectral features with spatial ones via a kernel function^31^, thereby transforming the linearly inseparable problem in low-dimensional space into a linearly separable one in high-dimensional space. The classification in high-dimensional space is realised by finding the best hyperplane, with the optimal results then mapped back to the dimension of the data to obtain the best classification solution.

Average accuracy refers to the average per-class effectiveness of a classifier. Micro F1 values indicate the relationship between a data point’s positive labels and those given by a classifier based on sums of per-text decisions, whereas macro F1 values reflect the relationship between a data point’s positive labels and those given by a classifier based on a per-class average^32^. In our study, most misclassifications occurred between samples at adjacent disease levels. This phenomenon can be explained by two factors. First, a single leaf may contain various forms of lesions whose areas differ from one another. Despite having the same disease level, a fluctuation in spectral reflectance still exists. Biological heterogeneity is another prime contributor to classification inaccuracy^33^. Such heterogeneity, which can occur within different leaves as well as within a single leaf, may result from the unbalanced distribution of water, solid matter, and air between veins and mesophyll^34^.

In another investigation of rice leaf blast, Yuan et al.^35^ used Savitzky–Golay (SG), standard normal variable, and multiplicative scatter correction algorithms to preprocess hyperspectral data. The SPA feature extraction method was combined with the SVM and linear discriminant analysis to construct separate rice leaf blast identification models. The SG-SPA-SVM model performed the best, achieving 98.7% accuracy.

Although we have demonstrated the feasibility of classifying rice leaf blast using STDs in this study, some problems still exist. First, the processing of hyperspectral images requires too much manual work, which impedes the inspection of large numbers of samples. Second, the experiment was conducted under a controlled environment, and extending these results to field conditions is still difficult. In the future, possible improvements to these two aspects will be investigated.

## Materials and methods

### Plant materials

In this study, we used Mongolian rice, a variety susceptible to rice blast. To more closely simulate the actual production situation, all test samples were directly collected from a naturally infected field located in Gengzhuang, China, at an experimental station of Shenyang Agricultural University (122° 73ʹ E, 40° 98ʹ N). The planting area was approximately 1000 m^2^. Rice seeds were acquired and placed in a seedling shed on 3 April 2021 for seedling cultivation and sown on 25 May. Urea–potassium sulphate–superphosphate fertiliser was basally applied at a rate of 270–80–130 kg/ha, with an additional application of 50 kg/ha urea performed at the tillering stage. To prevent insect pests from affecting the experiment, 5 g of chlorpyrifos 74% wettable powder (Shanghai Nongle Agricultural Chemical, Shanghai, China) was mixed with 10 kg of water, and this solution was sprayed with a T20 UAV system (SZ DJI Technology, Shenzhen, China) on 1 June, 1 July, and 1 August. The first symptoms of leaf blast were detected in early July, with a serious increase in disease observed approximately 1 week later. At each sampling time point, 12 clusters of diseased rice plants and 2 clusters of healthy ones were randomly selected from the field and transferred in a barrel (42 cm diameter and 50 cm depth) to a hyperspectral imaging room. Hyperspectral images of rice leaves were acquired the next day. The detailed description of samples was shown in Table 5.

**Table 5:** Description of samples used in this study.

Collection date	Growth stage	Sample	Training set	Testing set	Total
July 13, 2021	Jointing stage	Level 0	7	3	10
		Level 1	14	6	20
		Level 2	10	5	15
		Level 3	6	3	9
		Level 4	1	1	1*
		**Total**	38	18	55
July 27, 2021	Booting stage	Level 0	12	6	18
		Level 1	35	14	49
		Level 2	10	4	14
		Level 3	19	9	28
		Level 4	13	5	18
		**Total**	89	38	127
August 12, 2021	Heading stage	Level 0	11	4	15
		Level 1	31	14	45
		Level 2	14	6	20
		Level 3	24	10	34
		Level 4	16	7	23
		**Total**	96	41	137

*The sole level-4 sample was assigned to both sets simultaneously.

### Hyperspectral imaging

**Figure 4:**
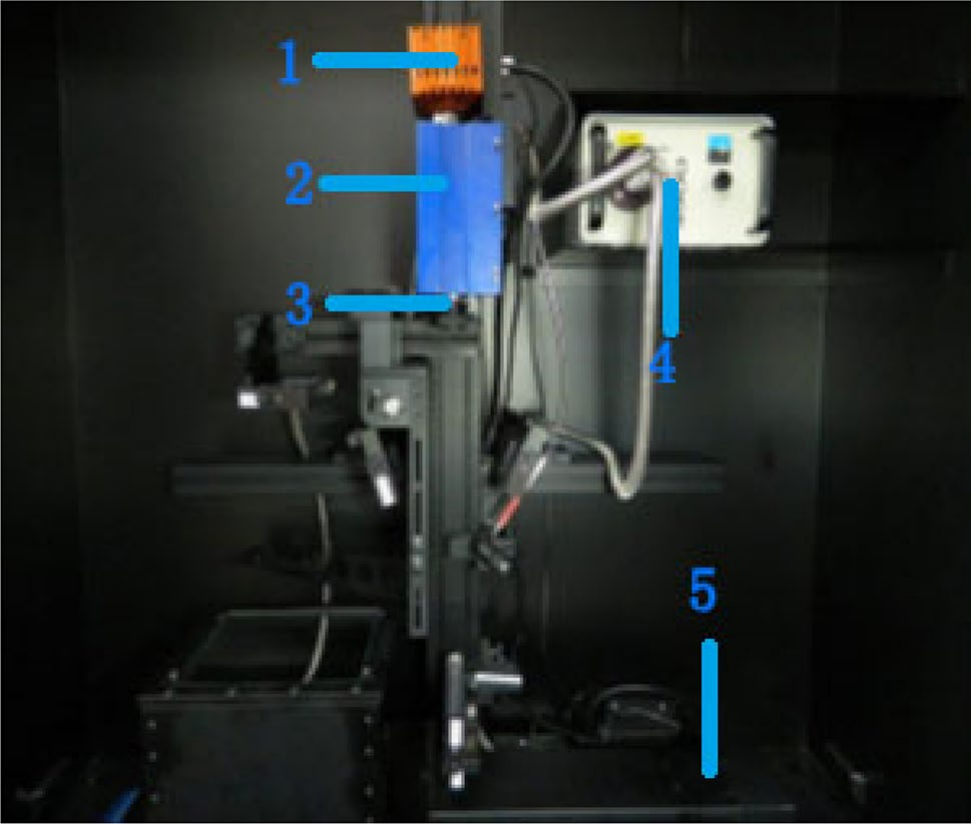
(1) High-sensitivity EM285CL EMCCD camera. (2) ImSpector V10E imager. (3) Camera lens. (4) IT 3900 halogen light source. (5) Scanning stage.

The imaging system used in this study (Fig. 4) consisted of a high-sensitivity EM285CL EMCCD camera (Raptor Photonics, Antrim, Northern Ireland), a stand-mounted ImSpector V10E imager (Spectral Imaging, Oulu, Finland), a horizontally adjustable scanning stage, a desktop computer with Spectral-Image software (Isuzu Optics, Hsinchu, China) for controlling the imager and scanning stage during image collection, and an IT 3900 halogen light source (Ocean Optics, Dunedin, FL, USA) to provide stable illumination^22^. The ImSpector V10E imager collected 472 wavebands over a spectral range of 400–1000 nm, encompassing visible-light and NIR regions, with a spatial resolution of approximately 1.27 nm. The angle of the left and right linear emitters was adjusted to a vertical orientation of 45° to enable the emitted light rays to converge on a coincident line just below the camera lens. The objective lens of the camera was set to an aperture of f/1.4. The distance between the camera lens and the scanning stage was set to 300 mm, and the focus was manually adjusted to guarantee image definition. The exposure time was manually adjusted according to the lighting environment to ensure sufficient incident radiation intensity. The speed of the scanning stage was set to 1.2 mm/s, with the aspect ratio set to the default. Leaves were carefully removed from each rice stem, placed flat on the stage, and gently affixed with double-sided adhesive. Five columns of rice leaves were placed parallel to one another on the scanning stage per imaging run. Great care was taken to avoid exerting pressure on the leaves. Any rice leaf exceeding 400 mm, the maximum sliding distance of the scanning stage, was cut into two or more sections while preserving the integrity of the diseased area. Spectra-Image software was used to capture images (4148 × 1024-pixel resolution), and the hyperspectral data cubes were saved onto an external hard drive.

Before further processing, the original hyperspectral images were corrected for dark current and uneven light intensity distribution after each scan^36^. First, a white board with high reflectance was scanned as a 100% standard. The value of Max DN was adjusted to 3600, which was 80% of the maximum value, and the bright field of the white board was recorded. The lens cap was covered, the dark field of the white board was measured, and the white board was then removed. Next, samples were situated directly below the camera on the scanning stage, with the exposure time adjusted to ensure Max DN was still at 3600 with the other parameters unchanged. After covering the lens cap, the dark field of the sample was recorded. The corrected sample image was calculated as follows:

1 $$R=\frac{{R}_{s}-{R}_{sd}}{{R}_{bw}-{R}_{bd}}$$

where *R* is the corrected sample image, *R*_*s*_ is the original hyperspectral image of the sample, *R*_*sd*_ is the hyperspectral image of the dark field of the sample, *R*_*bw*_ is the hyperspectral image of the bright field of the white board, and *R*_*bd.*_ is the hyperspectral image of the dark field of the white board.

### Classes of disease severity

Rice leaves were manually traced using the ROI tool in ENVI 5.6 (ITT Visual Information Solutions, Boulder, CO, USA). The area of the rice leaf was selected as a region of interest (ROI), and the number of pixels within it was counted automatically and recorded as N_1_. The number of pixels in diseased areas was calculated in the same way and recorded as N_2_. The degree of rice blast on a leaf was calculated as the percentage of the whole leaf area covered by lesions, that is, (N_2_ / N_1_) × 100%. Disease severity was then classified into six levels according to previously published standards^37^ as follows: 0, no visible lesions; 1, up to 2% of the leaf showing rice blast symptoms; 2, 2% to 5% showing symptoms; 3, 5% to 10% showing symptoms; 4, 10% to 20% showing symptoms; and 5, over 20% showing symptoms. Because samples at level 5 were only found under extremely severe disease conditions, samples from only five classes (levels 0 to 4) were discriminated in this study.

### Analysis of hyperspectral dataset

HSI Analyzer (Isuzu Optics, Hsinchu, China) was used to normalise hyperspectral images against known values of the white reference standard. A whole rice leaf was selected as a ROI and manually traced using the ROI tool. The average spectral reflectance, *R*_*w*_, was then extracted using the same tool. The STD of *R*_*w*_ at each wavelength was calculated as follows:

2 $$\mathrm{STD}=\sqrt{\frac{{\sum }_{j=1}^{n}{({R}_{j}-{R}_{w})}^{2}}{n-1}}$$

where *R*_*j*_ is the spectral reflectance of pixel *j*, *R*_*w*_ is the average spectral reflectance of the whole leaf, and *n* is the number of pixels contained in the whole leaf.

The acquired spectral reflectance consisted of two parts: true value and noise. Equation (2) can thus be expressed as:

3 $$\mathrm{STD}=\sqrt{\frac{{\sum }_{j=1}^{n}({R}_{Tj}+{R}_{Nj}-\frac{\sum_{j=1}^{n}{R}_{Tj}+{R}_{Nj}}{n})}{n-1}}$$

where *R*_*Tj*_ is the true value of the reflectance of pixel *j*, and *R*_*Nj*_ is the noise of pixel *j*.

Spectral noise has two components: air absorption and equipment noise. Air absorption is affected by the distance between pixels and the lens, whereas equipment noise is influenced by voltage. The width of rice leaves is only approximately 1 cm, which is roughly 1/30 of the object distance. As a result, the distribution of pixels is irrelevant when calculating pixel–lens distances. For the whole leaf, air absorption can be considered to be constant. In a single imaging run, the noise generated by the hyperspectral imaging system thus remains unchanged. Equipment noise also stays the same for the whole leaf. Overall, *R*_*Nj*_ is equal to $\frac{\sum_{j=1}^{n}{R}_{Nj}}{n}$ for a single leaf. Equation (3) can thus be expressed as:

4 $$\mathrm{STD}=\sqrt{\frac{{\sum }_{j=1}^{n}({R}_{Tj}-\frac{\sum_{j=1}^{n}{R}_{Tj}}{n})}{n-1}}$$

As can be seen from (4), the value of STD only depends on the true value of hyperspectral reflectance, thereby demonstrating its capacity in noise resistance.

### Construction of the support vector machine model

The STDs of spectral reflectance and raw spectral reflectance were classified into different degrees of disease severity by a non-linear SVM^38^. The classification obtained from STDs was compared with that from the raw spectral reflectance data, which served as the baseline accuracy. The applied SVM used the radial basis function as the kernel function to determine non-linear discriminant functions. As a supervised method, the SVM relied on training data. In this study, randomly selected samples were chosen as the training set, with the remaining assigned to the testing set. To build the optimal SVM model, the penalty parameter of the error term *C* and the kernel parameter *g* were optimised using a fivefold grid-search optimisation^39^. The range of *C* was set to 10^ N^ (− 10 ≤ *N* ≤ 10, with a step size of 0.1), and that of *g* was defined as 10^ M^ (− 15 ≤ *M* ≤ 5, with a step size of 0.1). The best penalty parameters were determined on the basis of the highest cross-validation accuracy of the training set. The performance of the SRR–SVM model was evaluated according to the average accuracy and micro and macro F1 scores of the testing set^32^. Model construction was carried out using LIBSVM 3.23^40^ (https://www.csie.ntu.edu.tw/~cjlin/libsvm/index.html) in MATLAB 2016b (MathWorks, Natick, MA, USA).

### Construction of the probabilistic neural network model

The STDs of spectral reflectance and raw spectral reflectance were also classified into different disease levels using a PNN model. The maximum number of iterations of the model was set to 100. To build the optimal PNN model, we varied the propagation speed *V* from 0 to 5 with a step size of 0.1 and selected the model with the highest classification accuracy. PNN model performance was evaluated using the average accuracy and micro and macro F1 scores of the testing set^32^. Data analysis and model construction were carried out in MATLAB 2016b.

The authors state that all methods were carried out in accordance with relevant guidelines.

## Data Availability

The datasets used and analysed during the current study are available from the corresponding author on reasonable request.
